# Association between sarcopenia and sleep disorders: a cross-sectional population based study

**DOI:** 10.3389/fnut.2024.1415743

**Published:** 2024-06-19

**Authors:** Kepeng Liu, Jinhui Luo, Yong Chen, Binfei Li, Ye Tian, Xianxue Wang, Xiaozu Liao

**Affiliations:** ^1^Department of Anesthesiology, Zhongshan City People's Hospital, Zhongshan, Guangdong, China; ^2^Department of Anesthesiology, The First Affiliated Hospital of Guangzhou Medical University, Guangzhou, Guangdong, China; ^3^Department of Anesthesiology, Changde Hospital, Xiangya School of Medicine, Central South University (The First People's Hospital of Changde City), Changde, Hunan, China

**Keywords:** sleep disorders, sarcopenia, sarcopenia index, cross-section study, NHANES

## Abstract

**Objective:**

Sleep disorders is a worldwide public health problem. We sought to examine the association between sarcopenia, a decline in skeletal muscle mass and function, and sleep disorders within the adult demographic of the United States during the period spanning 2011 to 2018.

**Methods:**

Diagnosis of sarcopenia and sleep disorders was ascertained through appropriate calculations and a structured questionnaire. The primary correlation analysis was conducted using a weighted multivariate logistic regression model. Furthermore, to confirm the presence of a potential non-linear association between sarcopenia and sleep disorders, additional analyses were performed using multivariate logistic regression and restricted cubic spline (RCS) regression with dose-response curve analysis. Subgroup analyses were also conducted to explore the influence of relevant socio-demographic factors and other covariates.

**Results:**

The final analysis encompassed 5,616 participants. Model 4, inclusive of all pertinent covariates, revealed a positive correlation between sarcopenia and sleep disorders, yielding an odds ratio (OR) of 1.732 (95% CI: 1.182–2.547; *P* = 0.002). Further analysis, utilizing the restricted cubic spline model, indicated a decreasing trend in sleep disorders as sarcopenia indices rose. Stratified analyses across diverse variables underscored the significant impact of sarcopenia on sleep disorders prevalence in several subgroups. Specifically, males, individuals aged 40 and above, non-Hispanic whites, those with high school education or equivalent, unmarried individuals, obese individuals (BMI ≥ 30), alcohol drinkers, former smokers, diabetics, and those engaging in less rigorous recreational activities exhibited a more pronounced association between sarcopenia and sleep disorders. The incidence of sleep disorders exhibited an upward trend as the incidence of sarcopenia declined among study participants.

**Conclusions:**

In summary, our study provides evidence of an association between sarcopenia and the prevalence of sleep disorders, with a negative correlation observed between the sarcopenia index and the odds ratio of sleep disorders. These findings suggest that maintaining optimal muscle mass may have a beneficial impact on sleep-related issues. In terms of exploring the mechanisms underlying the relationship between sarcopenia and sleep disorders, more in-depth research is warranted to ascertain the definitive causal relationship.

## Introduction

Sleep, a physiological process of paramount importance for our survival, assumes a vital role in facilitating reparative mechanisms for a wide array of potential injuries (1). Regrettably, inadequate sleep has become highly prevalent within contemporary society, with a substantial proportion of adults in the Americas, Europe, and Asia obtaining less than the recommended 7 h of sleep per night (2, 3). Multiple factors exert a substantial influence on sleep quality, encompassing gender, genetic predisposition, social dynamics, and geographical variations. In addition to insomnia, sleep-disordered breathing, central narcolepsy, circadian sleep-wake disturbances, parasomnia, and sleep-related movement disorders, the third edition of the International Classification of Sleep Disorders (ICSD-3) distinguishes distinct sleep disorder categories as well (4). These sleep disorders engender significant repercussions on both physical and mental wellbeing (5), fostering an augmented susceptibility to chronic ailments like hypertension, diabetes, obesity, and increased mortality rates (6). Consequently, conducting further comprehensive investigations into the multifaceted factors associated with sleep disorders proves both valuable and worthwhile.

The term sarcopenia is defined as “age-related muscle loss, affecting a combination of appendicular muscle mass, muscle strength, and/or physical performance measures (7).” While a decrease in muscle mass is a defining characteristic, it is crucial to recognize that sarcopenia encompasses the broader decline in muscular function, including strength and physical performance. An individual suffering from sarcopenia will experience a progressive decline in muscle mass and function, as well as a variety of health problems (8). Extant literature reveals that the prevalence of sarcopenia can escalate to 18% in individuals with diabetes (9), and similarly, patients undergoing surgery for kidney and liver diseases as well as those afflicted by certain cancers display a heightened incidence of sarcopenia across different anatomical locations (10–12). Notably, sarcopenia is also linked to several dysmetabolic conditions, including liver cirrhosis. There is compelling evidence indicating that 33% of individuals with liver cirrhosis manifest sarcopenia (13). Moreover, sarcopenia has exhibited strong associations with cognitive impairment, functional deterioration, hospitalization, hypertension, and depression within general populations (8). Although prior investigations have established a positive link between both insufficient and excessive sleep durations and the risk of sarcopenia (14), there remains a dearth of studies exploring the potential association between sleep disorders, such as poor sleep quality and insomnia, and sarcopenia. Accordingly, the objective of the current investigation is to scrutinize the correlation between sarcopenia and sleep disorders within a cohort that is nationally representative, employing data acquired from the esteemed National Health and Nutrition Examination Survey (NHANES).

## Materials and methods

### Study design and participants

This retrospective study utilized the data collected during the 2011–2018 cycle of the National Health and Nutrition Examination Survey (NHANES). The study protocol underwent meticulous scrutiny and obtained the necessary approval from the NHANES institutional review board (IRB) to ensure compliance with ethical standards. Furthermore, informed consent was duly obtained from all participants prior to their inclusion in the study.

**Figure 1:**
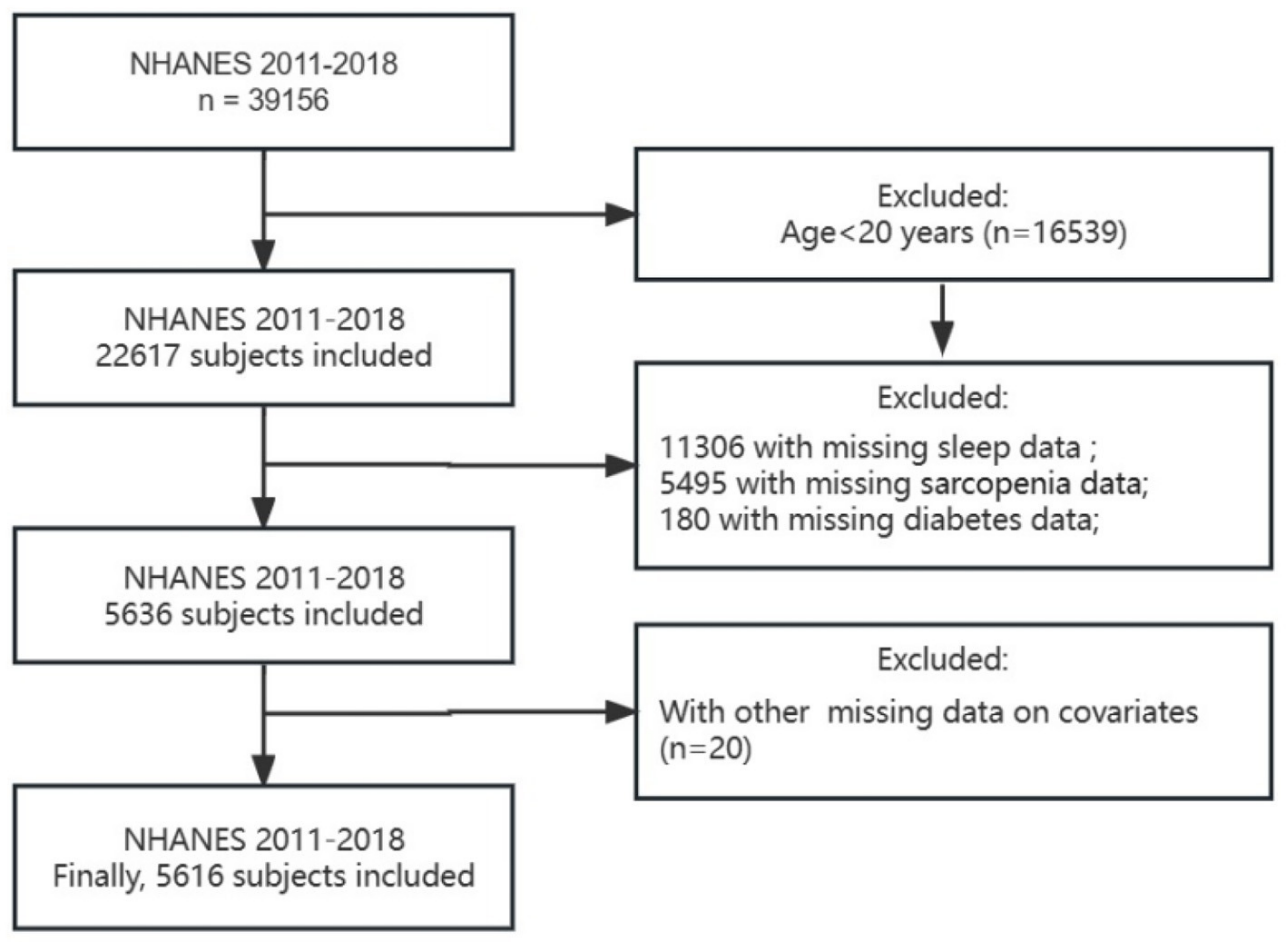
Schematic flow diagram of inclusion and exclusion criteria.

For the present study, a total of eight cycles of NHANES data were selected, encompassing an initial participant pool of 39,156 individuals. Subsequently, several exclusion criteria were applied, leading to the removal of participants meeting the following conditions: (1) Age below 20 years (*n* = 22,617); (2) Absence of sleep disturbance information (*n* = 11,288); (3) Insufficient data on sarcopenia (*n* = 5,504); (4) Insufficient data on diabetes (*n* = 180); (5) Missing information regarding age, sex, marital status, BMI, education, and smoking status (*n* = 20). Ultimately, a cohort of 5,616 participants was included in the study (Figure 1).

### Exposure variable and outcomes variable

The diagnosis of sarcopenia is facilitated through the utilization of dual-energy X-ray absorptiometry (DXA) scanning. The precision offered by the DXA scan in measuring both muscle mass and strength renders it an exceptionally accurate method for diagnosing sarcopenia. DXA scans were conducted on participants up to the age of 59 using a Hologic Discovery model A densitometer (Hologic, Bedford, MA, United States). The primary exposure variable in this study focused on sarcopenia, which was primarily assessed by aggregating the muscle mass of the four limbs, known as appendicular lean mass (ALM). In formulating our criteria for sarcopenia, we drew upon relevant guidelines while also incorporating the utilization of the sarcopenia index formula: total appendicular skeletal muscle mass (in kilograms) divided by body mass index (in kilograms per square meter) (15). Sarcopenia was diagnosed if the calculated index value for men was below 0.789 and for women was below 0.51213 (16).

The major outcome variable of interest in this study pertained to sleep disorders. Data pertaining to sleep disorders were obtained from the NHANES database through the administration of questionnaires. Throughout a continuous span of four NHANES cycles conducted from 2011 to 2018, a standardized questionnaire methodology was consistently implemented. Participants were inquired about their sleep disorders through a dedicated sleep disorder questionnaire, particularly with the question, “Have you ever been informed by a medical doctor or another healthcare professional that you have a sleep disorder?” This query served as a means to ascertain the presence or absence of sleep disorders. Responses such as “Do not know” or “Refused” were indicative of missing data regarding the existence of sleep disorders. By consistently posing this specific question across all four cycles, the study ensured consistency in data collection and facilitated a comprehensive assessment of the prevalence of sleep disorders among the participants.

### Covariate assessment

In accordance with previous research, this study incorporated pertinent covariates encompassing sociodemographic and lifestyle factors. Sociodemographic characteristics included sex (female, male); age (<40 years, ≥40 years); race (non-Hispanic white, non-Hispanic black, Other/multiracial, Mexican American, and other Hispanic); Education level (less than high school, high school or equivalent, college or above); Marital status (married, unmarried); body mass index (underweight, normal, overweight, obese). Lifestyle characteristics encompassed alcohol use (no/unknown, yes);smoke status (never, former, current); diabetes (yes, no), defined as a self-reported doctor diagnosis, glycated hemoglobin A1c (HbA1c) ≥ 6.5%, usage of insulin or anti-diabetes medications, fasting glucose ≥ 7.0 mmol/L, random glucose ≥ 11.1 mmol/L, or an oral glucose tolerance test (OGTT) ≥ 11.1 mmol/L (17).; vigorous recreational activities (no/unknown, yes); and moderate recreational activities (no/unknown, yes).

### Statistical analysis

To account for the intricate survey design factors, including sample weights, stratification, and clustering, appropriate weighting procedures were implemented in accordance with the NHANES analytical guidelines. Continuous variables were reported as the mean with standard deviation (±SD), whereas categorical variables were represented as percentages (%). Logistic regression analysis was conducted to determine the odds ratio (OR) and their corresponding 95% confidence intervals (CI), providing insights into the associations between sarcopenia and sleep disorders.

In the extended analysis, multiple models were developed to evaluate the relationship between sarcopenia and sleep disorders while accounting for various covariates. Model 1 presented unadjusted results. In model 2, baseline age, sex, and race were incorporated as adjustment variables. Model 3 further incorporated additional adjustments for education, marital status, and body mass index (BMI). Furthermore, model 4 included adjustments for alcohol use, smoking status, diabetes, vigorous recreational activities, and moderate recreational activities.

To depict the non-linear relationship, we employed the powerful tool of restricted cubic spline (RCS) functions, allowing for a comprehensive characterization of the dose-response relationship between sarcopenia and sleep disorders. Statistical analyses were conducted using R, version 4.2.3 (R Project for Statistical Computing). Statistical significance was determined with a threshold of *P* < 0.05.

## Results

### Patient characteristics

Table 1 presents the fundamental characteristics of the 5,616 participants recruited from the NHANES dataset spanning 2011–2018. Among these participants, 439 (8.6%) were identified as having sleep disorders. No statistically significant differences were observed in terms of sex, marital status, and alcohol use between the two groups (*P* > 0.05). However, significant differences were identified in several other characteristics, such as age, race, education, BMI (kg/m^2^), smoking status, diabetes, sarcopenia, vigorous recreational activities, and moderate recreational activities (*P* < 0.05).

**Table 1:** Weighted characteristics of participants in the NHANES (2011–2018) by sleep disorder.

**Characteristic**	**Overall, *N* = 5,616 (100%)**	**No sleep disorder, *N* = 5,177 (91%)**	**Sleep disorder, *N* = 439 (8.6%)**	***P*-value**
Age (years)	40 (29, 50)	39 (29, 49)	46 (35, 53)	**<0.001**
**Sex**				0.4
Female	2,790 (49%)	2,568 (49%)	222 (47%)	
Male	2,826 (51%)	2,609 (51%)	217 (53%)	
**Age**				**<0.001**
<40 years	2,849.0 (48.9%)	2,697.0 (50.4%)	152.0 (32.3%)	
≥40 years	2,767.0 (51.1%)	2,480.0 (49.6%)	287.0 (67.7%)	
**Race**				**<0.001**
Non-Hispanic White	2,179 (64%)	1,936 (63%)	243 (75%)	
Non-Hispanic Black	1,208 (11%)	1,119 (11%)	89 (9.8%)	
Other/multiracial	987 (8.6%)	950 (9.0%)	37 (4.8%)	
Mexican American	725 (10.0%)	690 (10%)	35 (5.5%)	
Other Hispanic	517 (6.6%)	482 (6.8%)	35 (4.7%)	
**Education**				**0.037**
Less than high school	1,014 (14%)	939 (14%)	75 (12%)	
High school or equivalent	1,193 (21%)	1,085 (20%)	108 (27%)	
College or above	3,409 (65%)	3,153 (66%)	256 (61%)	
**Marital status**				0.6
Married	2,720 (52%)	2,521 (52%)	199 (54%)	
Unmarried	2,896 (48%)	2,656 (48%)	240 (46%)	
**BMI (kg/m** ^**2**^ **)**				**<0.001**
Underweight (<18.5)	99 (1.5%)	94 (1.4%)	5 (2.1%)	
Normal (18.5 to <25)	1,738 (30%)	1,673 (32%)	65 (15%)	
Overweight (25 to <30)	1,787 (34%)	1,680 (34%)	107 (27%)	
Obese (30 or greater)	1,992 (35%)	1,730 (33%)	262 (55%)	
**Alcohol use**				0.075
No/unknown	1,028 (14%)	972 (15%)	56 (11%)	
Yes	4,588 (86%)	4,205 (85%)	383 (89%)	
**Smoking status**				**0.003**
Current smoker	1,324 (23%)	1,190 (22%)	134 (29%)	
Former smoker	946 (19%)	855 (19%)	91 (23%)	
Never smoker	3,346 (58%)	3,132 (59%)	214 (47%)	
**Diabetes**				**<0.001**
Yes	463 (8.3%)	388 (7.5%)	75 (17.1%)	
No	5,153 (91.7%)	4,789 (92.5%)	364 (82.9%)	
**Sarcopenia**				**<0.001**
Yes	430 (7.7%)	366 (7.1%)	64 (14.6%)	
No	5,186 (92.3%)	4,811 (92.9%)	375 (85.4%)	
**Vigorous**				**<0.001**
No/unknown	3,972 (69%)	3,618 (68%)	354 (82%)	
Yes	1,644 (31%)	1,559 (32%)	85 (18%)	
**Moderate**				**0.001**
No/unknown	3,128 (53%)	2,852 (52%)	276 (63%)	
Yes	2,488 (47%)	2,325 (48%)	163 (37%)	

The bold values represent *P* < 0.05, indicating statistical significance in the analysis results.

Furthermore, Table 2 presents the baseline characteristics of participants, with 430 individuals (7.2%) diagnosed with sarcopenia. Significantly divergent profiles were observed between the no-sarcopenia group and the sarcopenia group regarding age, race, education, BMI (kg/m^2^), alcohol use, diabetes, sleep disorders, vigorous recreational activities, and moderate recreational activities (*P* < 0.05).

**Table 2:** Weighted characteristics of participants in the NHANES (2011–2018) by sarcopenia.

**Characteristic**	**Overall, *N* = 5,616 (100%)**	**No sarcopenia, *N* = 5,186 (93%)**	**Sarcopenia, *N* = 430 (7.2%)**	***P*-value**
Age (years)	40 (29, 50)	40 (29, 49)	46 (35, 55)	**<0.001**
**Sex**				0.3
Female	2,790 (49%)	2,565 (49%)	225 (47%)	
Male	2,826 (51%)	2,621 (51%)	205 (53%)	
**Age**				**<0.001**
<40 years	2,849.0 (48.9%)	2,693.0 (49.9%)	156.0 (35.4%)	
≥40 years	2,767.0 (51.1%)	2,493.0 (50.1%)	274.0 (64.6%)	
**Race**				**<0.001**
Non-Hispanic White	2,179 (64%)	2,040 (64%)	139 (55%)	
Non-Hispanic Black	1,208 (11%)	1,175 (12%)	33 (4.1%)	
Other/multiracial	987 (8.6%)	916 (8.6%)	71 (8.6%)	
Mexican American	725 (10.0%)	602 (9.0%)	123 (22%)	
Other Hispanic	517 (6.6%)	453 (6.3%)	64 (11%)	
**Education**				**<0.001**
Less than high school	1,014 (14%)	894 (13%)	120 (23%)	
High school or equivalent	1,193 (21%)	1,082 (20%)	111 (25%)	
College or above	3,409 (65%)	3,210 (66%)	199 (52%)	
**Marital status**				0.7
Married	2,720 (52%)	2,496 (52%)	224 (51%)	
Unmarried	2,896 (48%)	2,690 (48%)	206 (49%)	
**BMI (kg/m** ^**2**^ **)**				**<0.001**
Underweight (<18.5)	99 (1.5%)	96 (1.5%)	3 (1.0%)	
Normal (18.5 to <25)	1,738 (30%)	1,699 (32%)	39 (5.2%)	
Overweight (25 to <30)	1,787 (34%)	1,688 (35%)	99 (22%)	
Obese (30 or greater)	1,992 (35%)	1,703 (32%)	289 (72%)	
**Alcohol use**				**<0.001**
No/unknown	676 (9.8%)	580 (9.0%)	96 (20%)	
Yes	4,588 (90%)	4,277 (91%)	311 (80%)	
**Smoking status**				0.058
Current smoker	1,324 (23%)	1,254 (23%)	70 (17%)	
Former smoker	946 (19%)	858 (19%)	88 (23%)	
Never smoker	3,344 (58%)	3,072 (58%)	272 (60%)	
**Diabetes**				**<0.001**
Yes	463 (8.3%)	385 (7.5%)	78 (18.2%)	
No	5,153 (91.7%)	4,801 (92.5%)	352 (81.8%)	
**Sleep disorder**				**<0.001**
No sleep disorder	5,177 (91%)	4,811 (92%)	366 (83%)	
Sleep disorder	439 (8.6%)	375 (7.9%)	64 (17%)	
**Vigorous**				**<0.001**
No/unknown	3,972 (69%)	3,592 (68%)	380 (90%)	
Yes	1,644 (31%)	1,594 (32%)	50 (10%)	
**Moderate**				**0.006**
No/unknown	3,128 (53%)	2,856 (52%)	272 (63%)	
Yes	2,488 (47%)	2,330 (48%)	158 (37%)	

The bold values represent *P* < 0.05, indicating statistical significance in the analysis results.

### Sarcopenia and sleep disorders

As demonstrated in Table 3, a notable and consistent association was detected between sarcopenia and sleep disorders. Across all four models, sarcopenia consistently displayed a positive correlation with the odds ratio of sleep disorders [Model 1: OR = 2.450, 95% CI: (1.719–3.449), *P* < 0.001; Model 2: OR = 2.493, 95% CI: (1.734–3.582), *P* < 0.001; and Model 3: OR = 1.756, 95% CI: (1.231–2.446), *P* < 0.001; Model 4: OR = 1.732, 95% CI: (1.182–2.547), *P* = 0.002]. Remarkably, no statistically significant difference in the odds ratio was found across all four models for the population aged <40 years.

**Table 3:** Logistic regression analyzed the relationship between sarcopenia and the presence of sleep disorder.

**Characteristic**	**Model 1**	**Model 2**	**Model 3**	**Model 4**
	**OR (95% CI)**	**OR (95% CI)**	**OR (95% CI)**	**OR (95% CI)**
**All participants**				
Sarcopenia				
No	Reference	Reference	Reference	Reference
Yes	2.450 (1.719–3.449)	2.493 (1.734–3.582)	1.756 (1.231–2.446)	1.732 (1.182–2.547)
*P* for trend	<0.001	<0.001	<0.001	0.002
**Age**<**40 years**				
Sarcopenia				
No	Reference	Reference	Reference	Reference
Yes	1.521 (0.667–3.431)	1.763 (0.791–3.943)	1.271 (0.590–2.743)	1.331 (0.652–2.721)
*P* for trend	0.300	0.150	0.500	0.400
**Age** ≥**40 years**				
Sarcopenia				
No	Reference	Reference	Reference	Reference
Yes	2.531 (1.682–3.793)	2.794 (1.848–4.202)	1.928 (1.251–2.977)	1.862 (1.163–3.014)
*P* for trend	<0.001	<0.001	0.002	0.005

**Figure 2:**
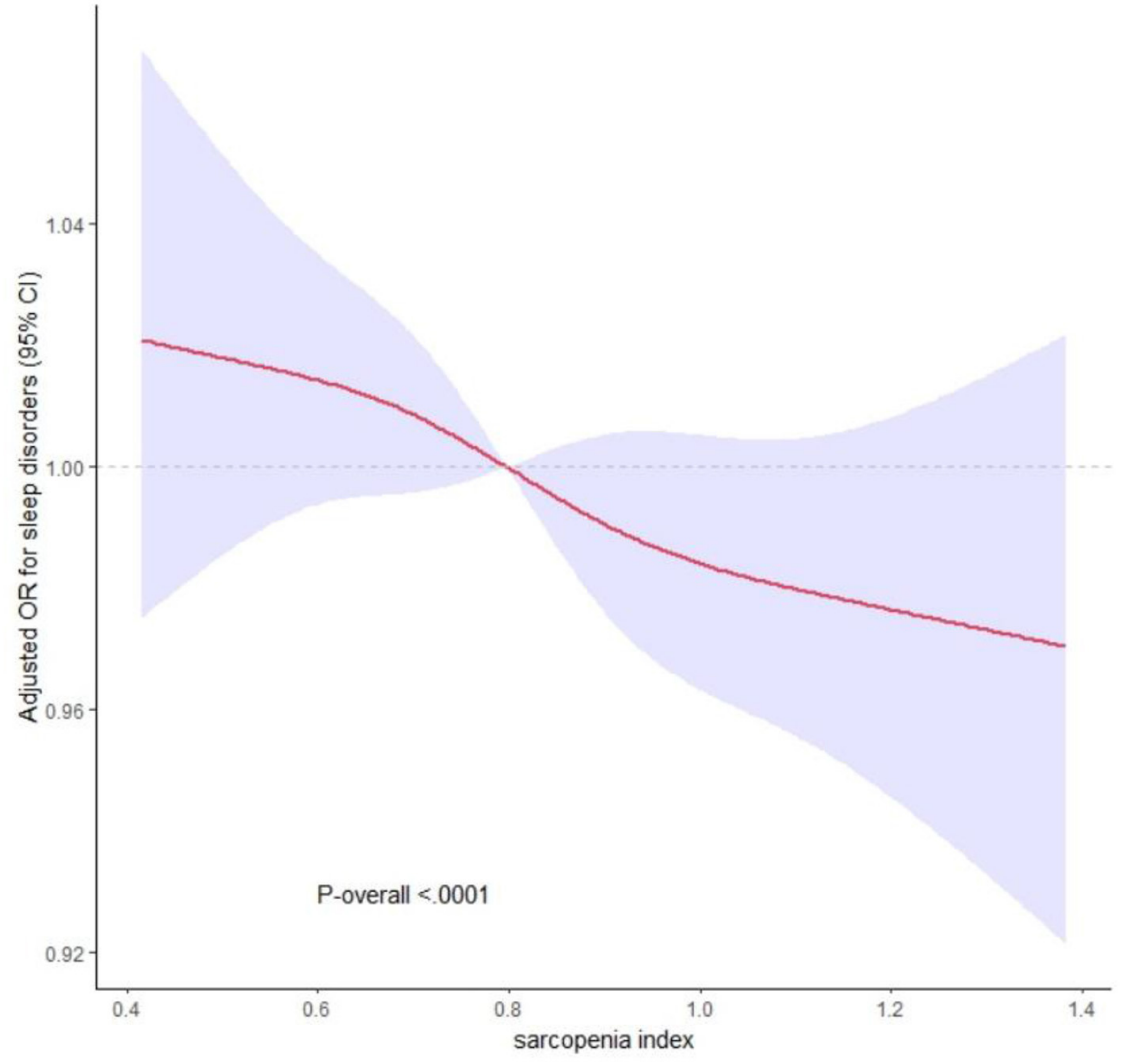
The dose-response relationship between sarcopenia index and sleep disorders. The solid and dashed lines represent the odds ratios and 95% confidence intervals. Adjustment factors are as same as which presented in extended model 4.

In the population aged ≥40 years, sarcopenia demonstrated a positive correlation with the odds ratio of sleep disorders [Model 1: OR = 2.531, 95% CI: (1.682–3.793), *P* < 0.001; Model 2: OR = 2.794, 95% CI: (1.848–4.202), *P* < 0.001; and Model 3: OR = 1.928, 95% CI: (1.251–2.977), *P* = 0.002; Model 4: OR = 1.862, 95% CI: (1.163–3.014), *P* = 0.005]. Subsequent analysis utilizing the restricted cubic spline model, built upon Model 4, unveiled a notable inverse relationship between the sarcopenia index and the incidence of sleep disorders. Notably, when the sarcopenia index reached 0.795, the odds ratio was observed to be <1, as depicted in Figure 2.

### Subgroup analysis

**Figure 3:**
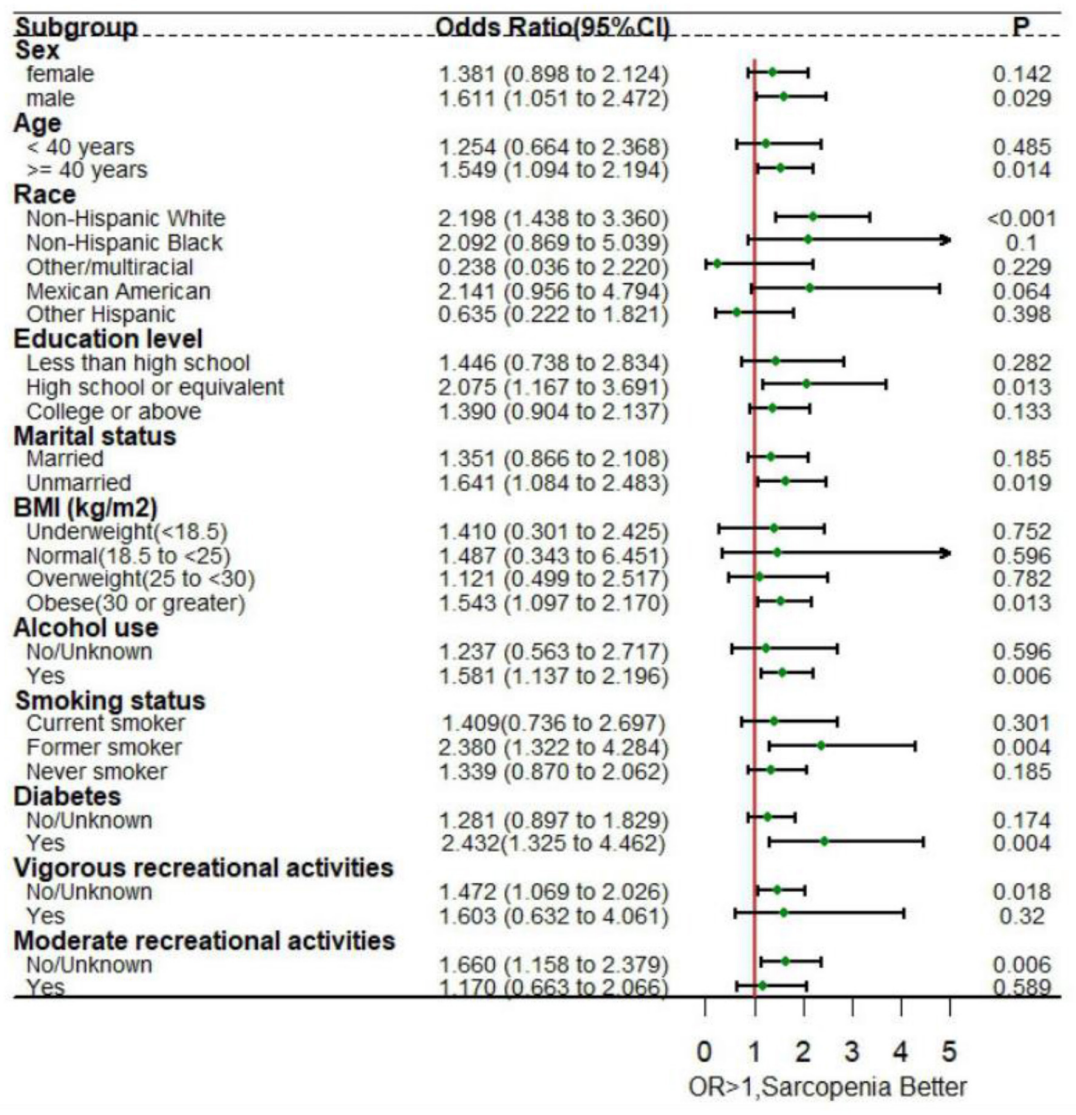
Forest plot for subgroup analysis of sarcopenia and sleep disorders. Sex, age, race, education level, marital status, body mass index, alcohol use, smoking status, diabetes, vigorous recreational activities, and moderate recreational activities were adjusted in the subgroup analysis.

Subgroup analysis was performed to assess the consistency of the positive association between sarcopenia and sleep disorders across different demographic groups. Stratified analysis based on different variables revealed that sarcopenia had a significant impact on the incidence of sleep disorders in several subgroups. Specifically, among males [OR = 1.611, 95% CI: (1.051– 2.472), *P* = 0.029), age≥ 40 years (OR = 1.549, 95% CI: (1.094–2.194), *P* = 0.014], non-Hispanic white person [OR = 2.198, 95% CI: (1.438–3.36), *P* < 0.001], those with a high school or equivalent education [OR = 2.175, 95% CI: (1.167–3.691), *P* = 0.013], unmarried individuals [OR = 1.641, 95% CI: (1.084–2.483), *P* = 0.019], those classified as obese [BMI ≥ 30; OR = 1.543, 95% CI: (1.097–2.17), *P* = 0.013], alcohol drinkers [OR = 1.581, 95% CI: (1.137–2.196), *P* = 0.006], former smokers [OR = 2.38, 95% CI: (1.322–4.284), *P* = 0.004], individuals with diabetes [OR = 2.432, 95% CI: (1.325–4.462), *P* = 0.004], those engaging in less vigorous recreational activities [OR = 1.472, 95% CI: (1.069–2.026), *P* = 0.018], and those participating in less moderate recreational activities [OR = 1.66, 95% CI: (1.158–2.379), *P* = 0.006], A more pronounced correlation between sarcopenia and sleep disorders was observed, as depicted in Figure 3. Additionally, it was observed that as the incidence of sarcopenia decreased among participants, the occurrence of sleep disorders increased.

## Discussion

The diagnosis of sleep disorders in this study relied on the criteria of receiving professional confirmation from a doctor or other healthcare providers. This criterion was adopted to mitigate potential subjective biases that may arise from self-reported sleep disorders. Sleep disorders in this study encompassed a range of conditions, including sleep apnea, insomnia, and restless legs syndrome, as documented in the NHANES database. Among the participants included in the weighted analysis, 8.6% were diagnosed with sleep disorders. The relationship between sarcopenia and sleep disturbances was examined in this study. The results revealed a significant inverse association between the incidence of sleep disorders and the sarcopenia index, suggesting a dose-response pattern. This finding is further supported by the analysis of the dose-response curve. Furthermore, subgroup analysis was performed to validate and explore the specific relationships, taking into account various influencing factors. The physiological differences between men and women, including hormonal levels, muscle mass, and body fat distribution, may contribute significantly to the more pronounced association between sarcopenia and sleep disorders observed in males.

Previous studies in the field of sleep research have predominantly concentrated on the central nervous system, whereas there is a growing recognition of the influence of muscle on sleep health. Past studies have demonstrated the significance of insulin-like growth factor 1 (IGF-1) as a pivotal anabolic hormone in maintaining muscle mass (18), and it has been indicated that increasing IGF-1 levels can enhance sleep quality (19). Preserving muscle mass and strength holds promising potential for effectively preventing sleep disorders. Notably, Ehlen et al. discovered that skeletal muscle possesses regulatory capabilities over sleep, with mice exhibiting higher expression of the BMAL1 protein in muscle tissue demonstrating faster recovery from sleep deprivation (20). BMAL1 serves as a key clock control gene within the mammalian circadian clock gene network, regulating not only the sleep-wake cycle but also influencing behavioral performance (21, 22). Furthermore, investigations have revealed a heightened risk of sleep apnea in individuals with muscular dystrophy (73% prevalence) (23). Studies have also associated sleep duration with skeletal muscle loss or decline (24). For instance, Nakakubo et al. found that prolonged sleep duration was linked to an elevated risk of sarcopenia progression among older adults (25). Moreover, older adults with either short or long sleep durations exhibited an increased likelihood of developing sarcopenia (26). Our own study observed a considerably higher incidence of sleep disorders (14.6%) among subjects with sarcopenia, in comparison to those without sarcopenia (7.1%).

Gender and age are acknowledged as independent factors that exert influence on both muscle quality and sleep health. Prior investigations have documented gender disparities in the relationship between sleep duration and sarcopenia, with a notable correlation observed among women while no significant association was found among men (24, 26). However, our subgroup analysis focusing on sarcopenia revealed that men are significant influencers of both sarcopenia and sleep, aligning with the findings of previous research (25). Due to the low proportion (7.2%) of cases progressing to sarcopenia during the follow-up period among the included subjects, we were unable to assess the correlation between sleep disorders and sarcopenia progression by gender. Therefore, further investigations with longer follow-up periods or larger sample sizes are warranted to explore the relationships between sleep, sarcopenia, and sleep disorders, and to elucidate any discrepancies among existing studies. Age is another important influencing factor, as previous research has demonstrated that aging induces significant physiological and functional changes in circadian rhythm, as well as muscle structure and function (27). In our study, we also found that sarcopenia is an independent risk factor for sleep disorders in subjects aged 40 years and older.

According to the research findings, individuals with type 2 diabetes demonstrate significantly reduced muscle mass and strength compared to those without diabetes (28, 29). Skeletal muscle plays a crucial role as a primary target for insulin action, and the decline in muscle mass and strength can potentially worsen insulin resistance (30). Our study also revealed that sarcopenia was present in 18.2% of participants with diabetes, which is considerably higher than the prevalence of 7.5% in non-sarcopenic individuals. Additionally, sleep disturbances are more prevalent among patients with type 2 diabetes compared to those without diabetes (31), which aligns with our findings. Sleep plays a crucial role in maintaining normal endocrine function, as evidenced by the negative correlation between fasting and postprandial blood glucose levels and sleep quality (32). Another study involving 110 patients with diabetes mellitus demonstrated a significant association between overall sleep quality and muscle strength in individuals with poorly controlled glycemic levels. Poor sleep quality was linked to increased consumption of sweets and reduced plasma levels of arginine, citrulline, and ornithine (33). Furthermore, poor sleep quality can lead to alterations in leptin and ghrelin levels, prompting increased sweet consumption and activating brain regions associated with reward and hedonic functions (34, 35). Excessive consumption of sugary foods is also linked to an increased risk of obesity. In our study, we found a significant correlation between sarcopenia and sleep disorders specifically among obese patients with a BMI >30.

Prolonged alcohol consumption has been associated with increased sleep latency and frequent arousals, leading to a significant reduction in sleep quality over time (36). Furthermore, a higher level of physical activity has been closely linked to improved sleep quality, a finding that is supported by our analysis data. In the group with sleep disorders, the prevalence of sleep disorders was significantly lower, with 18% observed in the higher activity group compared to 82% in the inactive group. These results are consistent with previous cross-sectional studies, suggesting that physical activity has a positive impact on sleep quality (37). Previous research has demonstrated that physical exercise can mitigate oxidative stress and markers related to apoptosis in the cerebral cortex and cerebellum, while also improving mitochondrial activity (38).

Human studies have shown that exercise training can alleviate various detrimental effects resulting from insufficient sleep (39). In particular, exercise training has been shown to decrease the mRNA expression and/or protein content of proinflammatory cytokines TNF-α and IL-1β in the hippocampus, which are typically elevated due to sleep deprivation (40). Increased levels of proinflammatory factors have been shown to adversely affect muscle mass and function, leading to increased muscle catabolism (41). Both inactivity and sleep disturbances can contribute to the progressive loss of muscle mass and function (42).

Recognizing the importance of exercise in preserving muscle health, clinical practice guidelines from 2018 strongly recommend physical exercise as the primary treatment for sarcopenia (43). Regular exercise or physical activity, particularly resistance exercise aimed at increasing muscle mass, significantly reduces the risk of developing sarcopenia (44). However, the current definition of sarcopenia, which primarily relies on appendicular mass, fails to capture its multifaceted nature. A more comprehensive definition that incorporates assessments of muscle mass, strength, and physical performance is urgently needed to provide a more accurate assessment of sarcopenia and its implications on health and quality of life. Future research should strive to incorporate a comprehensive set of measures to fully capture the complexities of sarcopenia and its clinical consequences.

## Limitations

Our research relied on data obtained from a sizable and nationally representative population sample, obtained through the NHANES survey. The sampling approach employed by NHANES ensured random selection of participants, leading to a sample that effectively represents the entire population of the United States. However, it is important to acknowledge several limitations in our study. Firstly, given the cross-sectional nature of our research design, we are unable to establish causality definitively, and the potential for reverse causality cannot be entirely ruled out. Secondly, in the studies included, the evaluation of subjective sleep relied almost entirely on questionnaires. The absence of objective sleep measurements may lead to potential biases and could impact the reliability of our findings. Furthermore, sleep disorders were not classified into specific types, and the correlation between different types of sleep disorders and sarcopenia was not analyzed. Third, there is a clear need for a more comprehensive definition of sarcopenia that incorporates both muscle mass and measures of muscle strength and physical performance. Such a definition would provide a more accurate assessment of sarcopenia and its impact on health and quality of life. Future studies should aim to include a comprehensive set of measures to fully capture the multifaceted nature of sarcopenia and its clinical implications. Finally, multiple studies have reported a higher prevalence of sleep disorders and sarcopenia in older adults (45, 46). However, dual-energy X-ray absorptiometry in NHANES is not suitable for individuals aged 60 years and older. Consequently, there is a dearth of discussion regarding sleep issues and muscle conditions associated with sarcopenia in the elderly population. Nonetheless, our study has yielded meaningful and intriguing results, serving as an important foundation for further investigation and research. In terms of exploring the mechanisms underlying the relationship between sarcopenia and sleep disorders, more in-depth research is warranted to ascertain the definitive causal relationship.

## Conclusion

In summary, our study successfully identified a relationship between sarcopenia and the occurrence of sleep disorders, revealing a negative correlation between the sarcopenia index and the odds ratio of sleep disorders. Furthermore, we conducted a subgroup analysis of the subject data to examine the varying effects of each covariate on sleep disorders. Among individuals aged ≥ 40 years, sarcopenia emerged as an independent risk factor for sleep disorders. Additionally, our study identified several significant influencing factors for both sarcopenia and sleep disorders, including male gender, obesity, alcohol consumption, previous smoking, and diabetes mellitus. These factors were found to have a significant impact on the development and occurrence of both conditions. Thus, the findings of our study provide support for the recommendation of regular physical activity in the general population to maintain adequate muscle mass and ameliorate sleep-related issues.

## Data availability statement

Publicly available datasets were analyzed in this study. This data can be found here: https://www.cdc.gov/nchs/nhanes/index.htm.

## Ethics statement

The studies involving humans were approved by the NHANES Institutional Review Board (IRB). The studies were conducted in accordance with the local legislation and institutional requirements. The participants provided their written informed consent to participate in this study.

## Author contributions

KL: Conceptualization, Data curation, Formal analysis, Writing – original draft, Writing – review & editing. JL: Data curation, Writing – original draft. YC: Data curation, Formal analysis, Writing – review & editing. BL: Data curation, Formal analysis, Writing – original draft. YT: Formal analysis, Writing – review & editing. XW: Data curation, Formal analysis, Writing – review & editing. XL: Writing – original draft, Writing – review & editing.
